# Enabling rational gut microbiome manipulations by understanding gut ecology through experimentally-evidenced in silico models

**DOI:** 10.1080/19490976.2021.1965698

**Published:** 2021-08-30

**Authors:** Juan P. Molina Ortiz, Dale D. McClure, Erin R. Shanahan, Fariba Dehghani, Andrew J. Holmes, Mark N. Read

**Affiliations:** a School of Chemical and Biomolecular Engineering, Faculty of Engineering, The University of Sydney, Sydney, Australia; b Faculty of Engineering, Centre for Advanced Food Engineering, The University of Sydney, Sydney, Australia; c School of Life and Environmental Sciences, Faculty of Science, The University of Sydney, Sydney, Australia; d Charles Perkins Centre, The University of Sydney, Sydney, Australia; e School of Computer Science, Faculty of Engineering, The University of Sydney, Sydney, Australia

**Keywords:** Precision medicine, dietary intervention, gut microbial ecology, host–microbiome interactions, genome-scale modeling, agent-based modeling, microbial culturing, computational microbiology, systems biology

## Abstract

The gut microbiome has emerged as a contributing factor in non-communicable disease, rendering it a target of health-promoting interventions. Yet current understanding of the host-microbiome dynamic is insufficient to predict the variation in intervention outcomes across individuals. We explore the mechanisms that underpin the gut bacterial ecosystem and highlight how a more complete understanding of this ecology will enable improved intervention outcomes. This ecology varies within the gut over space and time. Interventions disrupt these processes, with cascading consequences throughout the ecosystem. *In vivo* studies cannot isolate and probe these processes at the required spatiotemporal resolutions, and *in vitro* studies lack the representative complexity required. However, we highlight that, together, both approaches can inform *in silico* models that integrate cellular-level dynamics, can extrapolate to explain bacterial community outcomes, permit experimentation and observation over ecological processes at high spatiotemporal resolution, and can serve as predictive platforms on which to prototype interventions. Thus, it is a concerted integration of these techniques that will enable rational targeted manipulations of the gut ecosystem.

## Introduction

A growing body of evidence implicates the gut ‘*microbiome*’, the complex ecosystem comprising the human gut and the microorganisms inhabiting it, as a contributing factor in the etiology of non-communicable diseases,^1–8^ thus positioning it as a potential therapeutic target. For instance, diet readily modulates the gut microbiome and could thus be used to intervene in microbiome–host interactions. However, whilst broad modulators of microbiome composition and metabolism are known, inter-individual variations complicate the design and targeting of beneficial interventions.^9,10^ People are unique organisms harboring individualized microbiomes, and their diverging intervention outcomes stem from variation in gut ecosystem constituents and processes. However, comprehensive understanding of the gut ecosystem is presently lacking and difficult to obtain. *In vivo* study lacks the necessary spatiotemporal sampling and observation capacity, and *in vitro* models cannot recapitulate the host’s complexity. Yet, they offer complementary perspectives, informing both mechanistic understanding of the microbiome’s influence on host health and cell-specific models. We propose that, by encapsulating this information, *in silico* models that enable the experimentation, insight, and predictive capacity needed to rationally design and target interventions are now possible. Whilst we focus primarily on bacterial taxa, as the most abundant and most studied portion of the microbiome, we note that archaea,^7^ eukaryotes,^8,11,12^ and viruses^6^ (e.g. phages) that co-inhabit the gut are gaining attention and are being found to also influence host health status.

**Figure 1.**
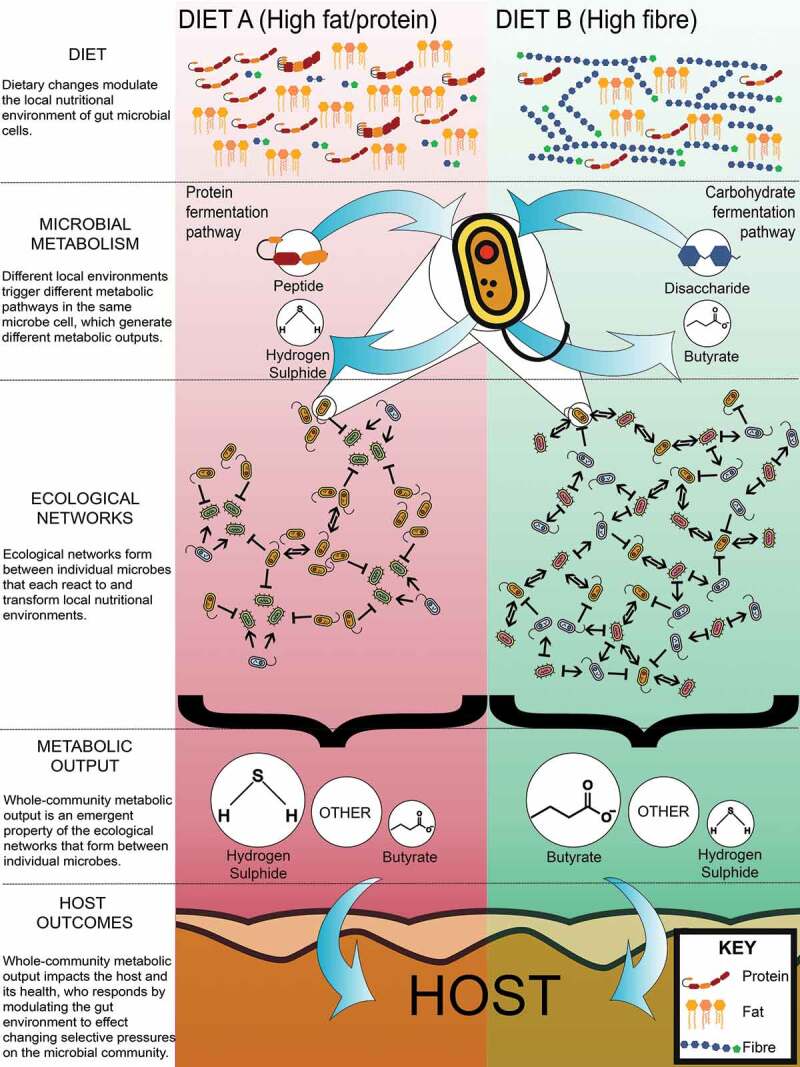
Health outcomes emerge from individual microbes and their survival strategies. Nutrient availability, primarily dietary, but also host secretions, drive microbes to regulate their metabolic capabilities to survive. Individual microbes adapting to their nutritional environment reconfigures the gut microbiome metabolic network and community-level metabolic output. The resultant changes can impact on host health

The microbiome’s impact on health outcomes is an emergent property manifesting from the collective activity of trillions of individual microbial cells vying for survival within their local gut environments (Figure 1). Interventions alter cell local environments and drive changes in cell behavior. These behavioral changes cascade through the community, reconfiguring strain niches and fitness, intercellular interactions, community metabolic output, and ultimately the functional responses of the host. The host integrates these signals, and its responses feedback on local microbial environments and alter selective pressures. Thus, host and microbial processes are intertwined and co-responsive. Given such interactive complexity, individualized and diverging responses to intervention are to be expected. Importantly, reasoning about the microbiome at the level of the strain (and thus cell) and it’s co-possession of numerous genes and metabolic pathways is essential: genes (and metabolic pathways) are only expressed when housed within viable organisms, and cell viability spans several nutritional requirements that involve crosstalk (coordination) between multiple metabolic pathways. Environmental perturbations need only limit a cell’s access to one nutrient to impact all its metabolic activities. As such, analyzing the microbiome through genes alone, independently of one another and the strains co-possessing them, will be of limited insight.

Two compositionally and functionally distinct microbiomes will adapt differently to a changing post-intervention environment and each host will respond differently to that adaptative process. Hence, to maximize effectiveness, interventions must be tailored to the individual. This ultimately entails (1) capturing the range of behaviors (‘dynamics’) each microbial strain in a community can exhibit across various environmental (including nutritional) contexts; (2) integrating these dynamics to extrapolate the resultant community-level outputs that can impact on host health; and (3) understanding host–microbiome interactions. Here, we consider how this can be achieved through an integration of modalities for studying the host-microbiome system.

*In vivo* studies associate host health outcomes with strains and molecular products, outcomes that originate from individual microbe-level behaviors. These factors form targets for control through intervention. *In vitro* culturing studies can reveal how specific strains respond to, and in turn modify, their environments. Understanding how and why interventions alter community-level emergent properties is not possible without detailed characterization of individual strain response dynamics. *In silico* modeling techniques deliver the integrative perspective of how cell-level behaviors scale up to the community-level phenomena that drive health outcomes. Models encode cell-level behaviors and replicate environment conditions, and then reveal spatiotemporal community outcomes.

### In vivo studies integrate whole gut ecosystem processes to highlight intervention targets

Non-communicable diseases are host organism-level properties manifesting, in part, from how microbiome and host interact to shape one another.^13^ *In vivo* study outcomes reflect an implicit integration of these factors. Of particular relevance is microbiome community composition. Host disease status has been associated with microbiome structural qualities including variation in strain relative abundances^14–19^ and/or microbial community diversity.^1–5,19,20^ Effects have occasionally been ascribed to individual strains: *Christensenella minuta* was found enriched in lean twins relative to their obese siblings, and inoculation of this strain into “obese” microbiomes transferred into mice reduced subsequent adiposity gains in recipient animals.^21^ However, isolating effects to specific microbial strains is difficult because strains overlap considerably in function. Yet it is pertinent to account for the effects of all strains, as an intervention may have no effect in a community if suppression of one strain elevates another of similar functional capacity.

Microbial community composition and metabolic output result from the growth substrates microbes have access to, and these primarily originate from host diet. Microbiomes shaped under high-fat and/or sugar diets have been associated with the etiology of diseases including diabetes,^22^ obesity,^22^ and hypertension.^23,24^ Conversely, dietary fiber consumption is associated with host health benefits,^25–27^ in part due to microbial fermentation of fiber into short chain fatty acids (SCFA).^28^ The direct dietary administration of SCFAs has conferred protection against induced colitis,^29^ food allergies,^30^ asthma,^31^ and diabetes^32^ in mice. However, associating diet–microbiome interactions with health outcomes is complicated. Firstly, microbial metabolic outputs vary with context. Substituting fiber with protein can increase microbial generation of pro-inflammatory metabolites, including hydrogen sulfide,^33,34^ ammonia,^35^ and phenolic compounds.^36,37^ Yet, protein fermentation can also lead to the generation of anti-inflammatory compounds such as butyrate^38^ and the polyamine agmatine.^39^ Secondly, diet is compositional: a high fat diet necessitates low protein and/or carbohydrate, so which nutrient confers a given effect? Systematic variation is required for parsing of effects. One such study showed interactive effects of macronutrients on microbiome community composition which in turn corresponded with host immunometabolism and body composition status.^40^ Dietary components are readily labeled as deleterious or beneficial to health, but in actuality effects are non-linear and wider context matters, yet this is difficult to account for.

Beyond diet, host-derived molecules including bile acids and mucus glycoproteins (mucins) secreted into the gut also impact the microbiome.^41,42^ Mucins shape mucosal microbiome communities as both a growth substrate^43^ and anchoring matrix^44^ for select commensals. For instance, *Akkermansia municiphila* and *Bacteroides thetaiotaomicron* can adhere to and hydrolyze mucin glycans and thus competitively colonize the gut mucosa.^44–47^ The result is that luminal and mucosal communities are compositionally^48^ and metabolically distinct,^49^ and as such these communities have different impacts on host health. The microbiome also metabolizes the primary bile acids cholic and chenodeoxycholic acids into numerous secondary bile acids that can actively regulate bacterial populations^50,51^ at phylum-^50^ and strain-levels.^52^ Together, host diets and endogenous secretions interact in shaping the microbiome, and rational intervention design should account for both, lest they present as confounding factors.

Targeted intervention design requires that we account for the effects of luminal and mucosal-associated strains on host health, and the various interacting dietary and host processes that shape the microbiome. Community and gut environmental contexts dictate which molecular products are produced, and what their contributions to health status are. *In vivo* study caries the advantage of integrating all such relevant factors in revealing host outcomes. Yet isolating specific causative factors is admittedly challenging as health status is rarely attributable to singular molecules and strains.

## Intervention targets are multiple, with broad host-microbiome molecular exchanges impacting health

*In vivo* research has revealed the complexity of the host–microbiome signal exchange that modulates health outcomes, which interventions should seek to manipulate. For instance, whilst the SCFA butyrate, as a primary colonocyte energy source, is protective against colorectal cancer,^53,54^ it also delays wound repair during overt inflammation or damage to the intestinal mucosa.^55^ Hydrogen sulfide, derived from bacterial metabolism,^56^ can accelerate the ulcer healing process observed in colitis,^57^ but also upregulate colorectal cancer cell division when compared to regular colonocytes.^58^ Single metabolites are not ubiquitously deleterious or beneficial. Again, the broader context matters, and targeted interventions must account for this.

Microbe-associated molecular patterns (MAMPs) are cell structural components that influence host inflammatory status. Classic examples include peptidoglycan, lipopolysaccharide (LPS), and flagellin. These molecules are structurally complex and can vary considerably between strains.^59–61^ Variants can modulate both innate^62^ and adaptive^63^ arms of the immune system to both pro-inflammatory and tolerogenic effects.^64–66^ For instance, LPS has been linked to the onset of obesity, diabetes, and cardiovascular disease.^67–69^ Yet, *Escherichia coli-*derived LPS decreased autoimmune diabetes incidence in animal models relative to *Bacteroides-*derived LPS.^70^ Thus, immune tone and disease status are a consequence of the balance of pro-and anti-inflammatory molecular signals.^71^ Interventions should seek to target not only single MAMPs but shape the holistic profile of molecules generated, and this requires understanding of the ecology through which community composition arises.

The host responds to microbiome signals to maintain homeostasis, but tipping points exist beyond which microbiome–host interactions devolve into perpetuating inflammation that compromises gut barrier function.^72^ A high-fat diet can induce this state by promoting overgrowth of gut bacteria with consequential over-production of pro-inflammatory cytokines by the host.^73^ Localized inflammation increases gut permeability, a condition known as *leaky gut*,^74^ followed by translocation of cytokines and MAMPs to portal circulation. Characterizing the boundaries at which tipping points transit between self-stabilizing beneficial and self-stabilizing deleterious host–microbiome interactions is critical, given interventions that seek to shift the ecosystem from one state into another.

Lastly, host–microbiome interactions vary both temporally and spatially within the gut. Being in closer proximity to host tissue, mucosa-associated strains interact more directly with the host, potentially exerting different and stronger pressures relative to those of luminal strains.^49^ Accounting for this mucosal gut microbiome is relevant; however, mucosa-associated strains are underrepresented in fecal samples, and this could distort the relationships inferred between observed microbial communities and their consequences on host health. Further, both microbial populations^75,76^ and environmental conditions^77,78^ are distributed heterogeneously along the colon, and undergo functional and compositional diurnal oscillations^79^ in part reflecting host feeding patterns.^42,80^ Detailed spatiotemporal characterization of such patterns is highly invasive and thus infeasible *in vivo*, but such heterogeneity is highly relevant to host healthfor example, the localized inflammatory regions characterizing Crohn’s disease.

In summary, *in vivo* studies reveal system tipping points whose breech negatively impacts the host. The profile of molecular interactions between host and microbiome underlie these phenomena but are spatiotemporally variable. Further, no single molecule is uniformly beneficial or deleterious: context is key. Controlling these interactions is non-trivial as microbiome molecular output stems from community-wide ecological interactions, and these are difficult to study *in vivo*.

## Complex community ecology underlies microbiome molecular output

Fermentative microbes have evolved a spectrum of survival strategies that rely on shorter/incomplete metabolic pathways^81^ that output metabolites such as SCFAs,^81–83^ that other microbes can further metabolize. A whole community engaging in such strategies yields complex inter-microbial dependencies. The profile of metabolites produced is difficult to forecast as it is sensitive to interactions among the microbial cells present, along with host diet and endogenous secretions. For instance, hydrogen sulfide can result from metabolism of endogenous and dietary cysteine and bile acids, thus its production depends on dietary protein^84^ and fat consumption,^33^ and the presence of sulfur-reducing organisms including *Clostridium* and *Enterobacter*.^33,34^

The capacity to generate most metabolites is widely distributed among phylogenetically diverse strains, and such redundancy further complicates metabolite output forecasting as has been observed for butyrate.^85,86^ Over 25% of strains, spanning several phyla, in a given individual’s microbiome can generate butyrate^87^ and four major butyrate production pathways with distinct substrates exist.^88^ Further illustrating the consequences of such redundancy, microbiomes sampled from three individuals varied in the profile of SCFAs produced when cultured using identical growth media.^89^ Thus, even in highly controlled culture conditions, a community’s possession of multiple pathways for generating a metabolite, and numerous strains supporting each pathway, render output prediction difficult.

Importantly, a strain’s presence and capacity for generating a given metabolite does not necessitate its actual engagement in the activity. Nutrient limitation will impact a cell’s capacity to grow. The nutritional environment is, in turn, modulated by host diet, which substrates metabolically active strains are presently consuming, and which intermediate metabolites they produce. Many microorganisms possess diverse strategies for satisfying their nutritional requirements. For example, *Bacteroides thetaiotaomicron* can degrade a wide range of complex dietary carbohydrates^90^ and host-derived glycans^91^ rendering it adaptive to varying nutritional contexts.^92^ Thus, accurate prediction of community composition and metabolic output necessitates accounting for cell nutritional (and thus growth) statuses, their nutritional context and how they modify it.

Any intervention that modulates a strain’s growth dynamics, in terms of prevalence and output, has the potential for cascading effects through the microbial community. Nutrient availability and competition dynamics shift, as will the profile of metabolites contributed back into the environment (Figure 1). These manifest as localized effects but can permeate more broadly through time and space. To rationally design interventions with predictable outcomes requires these ecological processes be characterized and their integrative consequences understood. This is extremely difficult to achieve in the real world, but possible *in silico* with support from *in vitro* studies that comprehensively map out strain-level growth dynamics.

## Comprehensive mapping of strain growth dynamics through in vitro study

The growth dynamics of individual strains, and their interactions with one another and specific host processes, can be characterized in detail outside of the living host. Environments reflecting locales in the gut can be imposed, controlling for nutritional context, water content, pH and selective inclusion of particular host factors. Strain growth rates, substrate utilization, and metabolic output can be quantified. *In vitro* technologies afford a comprehensive mapping of how given strains respond to changing environments. Thus, mucin and high pH were identified as fundamental requirements for *A. muciniphila* colonization, explaining its prevalence in the distal colon.^93,94^ Capturing strain growth requirements is essential for intervention design, as they determine whether or not a particular strain can grow given a particular nutrient context, with downstream implications for community composition and metabolic output.

How strains adapt their behaviors under changing environments likewise influences intervention outcomes. Microbial substrate preferences can impact competition dynamics and have been uncovered. For instance, *B. thetaiotaomicron* prioritizes mannose over other monosaccharides,^95^ and plant-derived polysaccharides over mucin carbohydrates.^96^ Similarly, lactate-utilizing bacteria prefer glucose over lactate when both are available.^97^ These substrate preferences are often strain-specific,^98^ reflecting differences in metabolic pathways. Strain metabolic output also differs with environmental context. Certain bacteria can generate formate, a common intermediate metabolite, but under specific pH conditions the same microorganisms can further metabolize it and generate gaseous hydrogen instead.^99^ In situations where formate-producing bacteria are paired with hydrogen-dependent microbes such as *Blautia hydrogenotrophica*, formate is also further metabolized to acetate and energy harvest is maximized.^100^ Even in pure culture, the relationship between the factors of substrate availability, cell capability/attributes and the outcome of net metabolite production is complex since they result from multiple causal (and interfering) pathways that vary over time.

Intricate metabolic interactions take place between gut microbes. Diverse ecological relationships, such as cross-feeding (mutualism), amensalism, and competition coexist in the gut to shape the microbiome and its metabolic output. *Cross-feeding* has been extensively studied owing to its scope for expanding a strain’s growth niche. For instance, in co-culture, *Ruminococcus bromii* produces formate which *B. hydrogenotrophica* consumes, and B. *hydrogenotrophica* reciprocates with panthothenate which *R. bromii* requires for optimal growth but cannot synthesize.^100^ This relationship also involves hydrogen utilization by *B. hydrogenotrophica* as described above. Concomitantly, *R. bromii* and *B. hydrogenotrophica* compete for the vitamin thiamine in this context, exemplifying how complex interactions in the gut can be. Microbes form a complex web of interactions and variations in microbial membership or activity can trigger a cascade of effects across the network. The units of biological activity in a community are not necessarily cells or strains – syntrophic dependencies mean multi-organism networks are more appropriate for modeling some outputs.

*In vitro* technological advancements are also facilitating detailed investigation of isolated host–microbiome interaction pathways. These methods cannot fully recapitulate the host but can elucidate the ‘behavioral building blocks’ from which whole-scale host–microbiome interactions emerge. Cell culture models employ specific cell lines to replicate the intestinal epithelium,^101^ capturing processes including mucin excretion, cell migration, and signaling. This has enabled study of bacterial adherence to epithelial cells and the former’s response to cytokine generation.^102^ Microfluidic technologies combined with cellular culturing have enabled the study of how peristalsis and intraluminal flow impact the gut microbiome.^103,104^ Multi-stage continuous fermentation models reveal spatiotemporal dynamics, e.g. that the impact of a dietary intervention was not equally distributed along the gut.^105^ For instance, the Simulator of the Human Intestinal Microbial Ecosystem (SHINE), a modular multi-stage continuous fermentation model, was extended to study mucosa-adherent microbiome dynamics (M-SHINE), specifically the recovery of *Lactobacillus* strains following an antibiotic pulse.^106^ Microbial-host interactions fundamentally shape microbial behaviors and *in vitro* approaches that trace and objectively measure them are important to rational intervention development. Advances in techniques to culture cells and measure physiology mean it is possible to effectively describe the range of cell states in response to variation in physio-chemical context (at least in much more detail than previously).^107^

These *in vitro* approaches represent ‘reductionist’ science: isolating and manipulating pathways and microbes to probe their dynamics. They offer insights that cannot be accurately generated through other means, but they also face limitations of scale. Complete characterization of strain dynamics necessitates wide, systematic exploration of environmental contexts and strain responses. It is impractical, even with advances in modern robotic fermentation systems, to conduct such broad experimentation for each of the thousands of strains occupying the gut. When compounded with the range of unique consortia that can be assembled and investigated, the experimental burden is staggering. For instance, to map the growth requirements around the eight B vitamins would entail 255 unique inclusion/exclusion combinations, which if applied to 400 strains (approximate richness of the human microbiome^108^) entails 102,000 cultures. A second key challenge is one of complexity; comprehensive characterization of strain dynamics is vital to understanding the principles of community organization and function, but there remains a sizable leap in scaling from these strain-level dynamics to predicting community outcomes. Reassembling and integrating these pathways to study how they deliver emergent behaviors and system-wide consequences is a ‘constructionist’ task. *In silico* techniques, fusing biological data with modeling, can accomplish this.

### *In silico* models: from individual microbes to community outcomes

*In silico* modeling relies on computer algorithms that recapitulate microbial and host dynamics. Such models integrate data from different modes of study and at varying biological scales, spanning metabolites, cells, communities, and the entire gut ecosystem with the aim of reproducing outcomes observed in real-world systems. In so doing, they seek to explain these emergent outcomes as manifesting from individual molecule- and cell-level dynamics. Validated models can be tremendously insightful: being computer code, they present no limits for intervention or spatiotemporal observation scope. Putative targets for intervention can be rapidly and systematically explored for their effects. Models can be used in a predictive capacity, exploring possible interventions to achieve a desired outcome.

Models can aid understanding of a system’s operation, elucidating the fundamental mechanistic principles underpinning the system through simplification to the most essential components and their interactions. Simplifying can mean modeling a subset of the full system, and can entail amalgamating distinct components (cells, molecules, pathways) with similar function or that constitute a module of given function (e.g. multi-organism networks) into a single-component types.^109^

Agent-based modeling (ABM) is one such ‘abstractive’ technology. ABMs can capture spatially explicit, heterogeneous environments occupied by discrete, dynamic agents whose interactions give rise to complex emergent outcomes.^109^ Modeled agents encode behavioral responses to environmental stimuli and interactions with one another which are dependent on an agent’s state: the result of its past experiences. With microbiome phylogenetic diversity exceeding functional diversity,^110^ findings reported in terms of phylogenetic taxonomies have become increasingly complicated to interpret.^111^ Interpretation through functional units and their interactions may prove more tractable and informative. ABM was used to abstract the gut microbiome into six trophic guilds that represented distinct nutritional strategies,^40^ thus distilling hundreds of stains into far fewer and functionally distinct terms. This model demonstrated that microbial strategies for acquiring nitrogen from either dietary or endogenous sources could explain the variation in community composition observed across a broad range of mouse diets. The model revealed ecosystem dynamics, explaining changes in community composition in terms of which nutrients were limiting the growth of each microbial cell; those cells’ nutritional environments as resulting from diet, host digestion, and endogenous substrate provision; and how effectively cells were competing for nutrients. Such mechanistic insight would have proven difficult to obtain through the lenses of phylogenetic and gene cataloging. This study also exemplifies the high-throughput of *in silico* modeling: 250 ‘mice’ administered varying diets were readily simulated within hours, whereas the corresponding *in vivo* work was a huge undertaking spanning many months.^40^ By offering unparalleled tracing of spatiotemporal dynamics, ABMs have shown how modifications in host epithelial secretions shift the microbial community^112^ and how isolated and aggregated feedback mechanisms (toxin-antitoxin, substrate sharing and antibiotic resistance dynamics) impacted gut microbiome resilience to common stressors (e.g. antibiotic therapy).^113^ These insights would have proven complicated to extract from real-world observations. Thus, ABMs can trace microbial dynamics to reveal the governing ecological principles at the single-cell level and what their community-level consequences are.

Not all modeling approaches strive for simplification, and models emphasizing capture of real-world detail seek to quantitatively explain real-world phenomena in those same terms. Genome Scale Models (GSMs) are one such technology. They are reconstructions of a cell’s genome-encoded metabolic pathways and networks,^114^ and simulations of communities are emerging.^115^ A common GSM application is to estimate a configuration of ‘fluxes’ (throughputs) through metabolic pathways that maximize organism growth rate(s) in a given metabolic environment: ‘flux balance analysis’.^116,117^ GSMs offer ease of systematic exploration of the nutritional dimension, allowing the sampling of hundreds of media formulations in a matter of hours. The technology can substitute for arduous *in vitro* culturing work to determine a strain’s nutritional growth requirements. For instance, an *A. muciniphila* GSM predicted the commensal could utilize a number of monosaccharides derived from mucin, findings that were subsequently validated *in vitro*.^118^ Similarly, GSMs have been used to identify the minimal media needed for *Faecalibacterium prausniitzii*^119^ and *Bacteroides caccae*,^120^ resulting in their successful culturing. Beyond determining strain niches, growth rates and metabolic output yields are potentially determinable through GSMs, but these are sensitive to bounds on nutritional uptake and biomass generation rates that are presently unknown for most strains. If these upper rate bounds can be determined through targeted *in vitro* cultures, and are otherwise invariant to nutritional context, then growth and metabolic output rates for very broad combinations of media could also be rapidly estimated *in silico* and thus save considerable *in vitro* effort.

GSMs can now be simulated in consortia and have provided insight into the ecological principles that underpin emergent community outcomes. This technology enables tracing of strain-level metabolic activities, and how their survival strategies vary with changing community contexts. For instance, using GSMs, a behavior was observed wherein one microbial guild’s reduction of growth rate (70% of its community-specific maximum growth rate) when paired with another guild allowed for an increased total community biomass, thus improving community-level fitness.^121^ Similarly, over 800 GSM microbial communities were assembled and assessed in terms of competition and cooperation dynamics and showed that whilst competition was the predominant interaction across the community, modules of up to four cooperating strains persisted across communities.^122^ The metabolites that were cooperatively exchanged were also identifiable.^122^ Such understanding is key for rational intervention design as it explains the underlying ecology that is difficult to otherwise interrogate through non-*in silico* means. Lastly, GSM consortia’s capture of community ecology has enabled successful prediction of intervention outcomes. This was accomplished for two groups of human patients differing in insulin resistance profiles;^123^ GSM consortia reflecting patient microbiomes correctly predicted changes in stool amino acid and SCFA levels under a dietary intervention that was subsequently administered to the humans. Thus, there is a potential role for GSM technology to guide intervention design and choice. Although useful, GSM technology caries some caveats, namely omitting genome regulatory networks and equating the genotype and phenotype. Yet, GSM technology is rapidly advancing,^114,124^ and is well poised to revolutionize our understanding of the gut ecology.

Recently, supervised machine learning has shown promise in predicting physiological outcomes (e.g., post-prandial glucose response^125^) of dietary manipulation, essentially treating microbiome–host interactions and dynamics as a ‘black box’. These approaches demonstrate that intervention outcomes can be predictable and therefore targeted at individuals. Yet they have limitations. These models are opaque and do not necessarily learn the true mechanisms underpinning the biology. Consequently, they may prove inaccurate in predicting outcomes for cases outside the training data’s range of intervention. Lastly, it is debatable how much penetration machine learning efforts will have if they are not accompanied by a well-understood mechanistic foundation.

*In silico* approaches offer continuous observation, at high spatiotemporal resolution, of the nutritional environment, cellular interactions, internal cell state and competition dynamics, and facilitate simulation of multifactorial interventions. They cannot (yet) simulate ‘health outcomes’, as these are emergent from the holistic host at a breadth current models do not capture. But they can model the profile of health-impacting community composition and metabolic output and the ecological dynamics responsible for them. GSMs are close to predicting these profiles for previously unobserved contexts, which would prove transformative for targeted intervention discovery.^123^ This could be accomplished through systematic exploration of intervention space (e.g. the space of possible dietary interventions). For example, a computer model of an individual’s gut microbiome (profiling of *in vivo* communities) could be constructed and used to predict the outcomes for an intervention based on the fiber inulin, accounting for: which strains can catabolize it (based on *in vitro* experimentation) and are they competitive to do so given the community; which metabolites will be generated from inulin based on this profile; how these metabolites will impact other strains and how this will ultimately impact the community’s composition and metabolic output. Such study is easily repeated with variation to investigate how outcomes relate, potentially non-linearly, with fiber dosage. To conclude, evidence-based *in silico* methods can offer unparalleled experimental resolution and traceability at the spatial-temporal and biological levels.

## Practical considerations for *in silico* modeling

ABM encompasses considerable flexibility in how the target biology is represented within the model, and this translates to models that require substantial custom computer code to implement. Which cells, pathways, molecules, and environmental features a given model captures is typically entirely bespoke to the given research context. Such flexibility complicates the generation of ‘one size fits all’ generalized simulation frameworks – lacking a standardization of what should be simulated and how, such frameworks would need to be vastly complicated in catering for all possibilities, and thus they instead typically represent very abstract and general tools that can require considerable custom code to adopt into a specific simulation.^126^ Our lab’s ARTIMMUS^127^ and MotiliSim^128^ agent-based models are built atop the MASON agent-based simulation framework,^129^ and yet encompass several thousand lines of custom Java code each. The GutSim simulation is entirely custom code, written in Python.^40^ Whilst existing ABM models can certainly be adapted to different research questions, engaging with the ABM paradigm can necessitate a considerable aptitude for writing computer code.

A key consideration for ABM technology, stemming from the flexibility it affords in which biological features are represented and how, is ensuring that ABM models correctly capture the biology and that they are well-calibrated against existing real-world data. By necessity of simulating a system that is incompletely understood, ABMs often encompass parameters that control agent behaviors for which appropriate values are not exactly known. Calibration aims to infer these values from existing data by exploring a range of putative parameter values with an aim of reproducing known results. Agent-based model calibration has been reviewed extensively elsewhere,^130^ and we summarize the salient points here. Calibration requires data describing target system behaviors, and there is no minimum threshold for this – more is better but with a diminishing return on additional data. Ideally, multiple real-world experiments will be used in calibration,^131^ as these perturb the real-world system (and thus the simulation in attempting to reproduce real-world results) in different ways, targeting various cells, pathways, genes, molecules, etc. This encourages the full range of each agent’s possible behaviors to be exercised and assessed. Thus, ideally, data or knowledge describing the behaviors, rates, probabilities, timings, etc., of each individual model component (e.g. strain) can be provided, but failing this, higher-level data (e.g. at community composition-level) describing how the biological system behaves both ‘normally’ and under perturbation can facilitate calibration efforts.

ABM models are not necessarily computationally expensive to run (Table 1). The scale of the simulation, in terms of number of agents (e.g. cells), size, complexity and detail of the physical simulated space can often be adjusted, and these are the chief determinants of how much computational power and time is needed for the simulations to execute. In our lab, we have often prototyped models on personal computers before deploying them on a high-performance computing (HPC) facility to execute many more replicates and experimental variations at a larger-scale than a personal computer could accommodate. Access to HPC facilities is rarely prohibitive these days, with most research institutes either providing them directly or facilitating access to shared utilities. Thus, the biggest resource needed in engaging with ABM technology is time and personnel in building the models. Relative to this, experimentation with the models is relatively swift and the software tools are either cheap or free to use. Model building is rarely done in a timeframe less than months, with calibration and exploration of which biological features to include, and how, taking the most time – writing code is easy, demonstrating and arguing that the code correctly captures the target biology is what takes time.^137^ This is an important (and easily overlooked) consideration, as without it results are misleading rather than insightful. Planning experiments to verify model predictions (whatever they may be; this is problem-specific) is a worthwhile activity to budget for and if successful can hugely elevate the work’s impact.

**Table 1.** List of recommended computational resources for gut microbiome in silico modeling

Resource	Summary	Available from
Personal computer	Most *in silico* based approaches can be ran on a basic personal computer. High specifications (e.g. 8GB of RAM or higher) are recommended to reduce modeling times	
High performance computing cluster	A network of fast interconnected computer servers that are more practical for running either: 1) very large scale simulations that a personal computer would struggle to handle or 2) very high-throughput experiments (many simulations) that a computing cluster can run in a massively parallel fashion.	Most universities and research institutes now provide such facilities at modest, if not zero, cost. Alternatively, cloud-based systems such as Amazon Web Services could be set up to support such work, though this often comes at a cost.
COBRA Toolbox^132^	MATLAB-based software suite for modeling genome-scale metabolic networks and predicting phenotypes	https://opencobra.github.io/cobratoolbox/stable/index.html
COBRApy^133^	Python package for modeling and analyzing genome-scale metabolic networks	https://opencobra.github.io/cobrapy/
MICOM^115^	Python package for metabolic modeling of microbial communities	https://github.com/micom-dev/micom
μbialSim	A dynamic Flux-Balance-Analysis-based simulator for complex microbial communities	https://git.ufz.de/UMBSysBio/microbialsim
AutoKEEGRec^134^	A KEGG databases-based tool to create draft GSMs and community reconstructions, that is compatible with COBRA Toolbox	https://almaaslab.nt.ntnu.no/index.php/resources/
ModelSEED^135^	A web resource to create GSMs from Rapid Annotation of microbial genomes using Subsystems Technology (RAST)	https://modelseed.org/
KBase^136^	Open source platform that allows the creation and curation of GSMs among other functions	https://kbase.us/applist/
AGORA^120^	A collection of 818 GSMs for human gut microbes compatible with COBRA Toolbox and COBRApy	https://www.vmh.life/#microbes/search
GutSim^40^	An ABM that integrates gut environmental pressures such as peristalsis, mucin secretion and host feeding regimens.	https://github.com/marknormanread/GutSim

Growing support around GSM has contributed a rich, accessible, and growing ecosystem of tools for the further development and application of this technology. Free online tools such as AutoKEGGReg^134^ and ModelSEED^135^ allow the generation of draft GSMs based on annotated genomes. Curated GSMs are commonly made freely available by researchers specialized in the matter, and the tools to simulate them are likewise freely accessible (Table 1). Curation can be the most time-consuming step of the GSM assembly process. GSMs are complicated, and manual critical inspection and verification against known organism behavior by a specialist cannot be avoided. Most readily available gut microbiome GSMs have been rigorously curated at a qualitative level of which metabolic reactions a given strain is capable of. However, we are not aware of any GSMs for which the upper bounds on reaction rates have been comprehensively calibrated. This is an important omission, as these reaction rate bounds ultimately constrain maximum growth rates, and thus community composition and metabolic output outcomes. Not knowing these upper bounds limits the extent to which GSMs can be used as faithful surrogates for real communities in predicting outcomes to putative interventions (i.e. where these outcomes are not already known and thus cannot be calibrated against). There is enormous upside in establishing these reaction rate limits, as the search for interventions that deliver targeted outcomes could then be automated and run in a massively parallel fashion on a HPC using GSM technology as faithful surrogates for the real community. Importantly, for a given GSM/strain, these rates could be inferred through a combination of *in vitro* culturing where specific growth rates, substrate uptake, and metabolite production rates can be established across a variety of media formulations, and through automated calibration methodologies that find parameter values that best recapitulate all these *in vitro-*observed strain behaviors simultaneously.^131^

GSM technology can be relatively straightforward to engage with. GSMs are built upon individual strain genomes, independently of one another, forming modules that can then be simulated individually or combined into communities in a ‘plug and play’ fashion. The COBRA^132^ and COBRApy^133^ toolboxes cater for individual GSM simulations, and frameworks such as MICOM^115^ and µbialSim^138^ support GSM simulations of communities. Coding proficiency in Python or MATLAB is required. Simulations of individual GSMs or simple consortia can be conducted on a personal computer, but HPC access is advisable for large communities or very broad experimental designs, such as extensive systematic exploration of nutritional variations or community memberships.

## Conclusion

**Figure 2.**
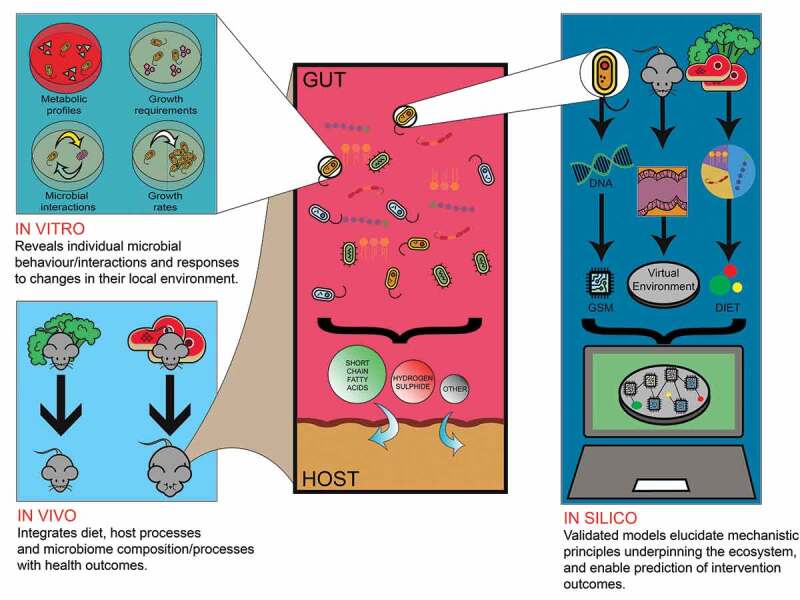
In vivo, in vitro and in silico methods each contribute complementary insights that are collectively necessary to understand gut ecology and manipulate it to achieve specific outcomes

The gut microbiome impacts host health and has emerged as a therapeutic target. The therapeutic value of currently available interventions, e.g. prebiotics, probiotics, or diet, is contingent on how they integrate within a host’s existing gut ecosystem.^9^ Yet this differs between hosts, and as such outcomes are divergent. Better design and targeting of interventions requires improved understanding of the ecological processes underlying microbiome composition and function. *In vivo* approaches have revealed the breadth and nature of interactions between host and microbiome; these are multiple and have non-linear, context-dependent effects. These interactions are a culmination of intertwined processes that shape both host and microbiome. However, *in vivo* approaches struggle to isolate and probe individual ecological processes at fine spatiotemporal resolution. Through *in vivo* approaches we understand which microbial outputs impact host health, but not why or how such outputs emerge as key factors across differing contexts and individuals. *In vitro* approaches permit isolation and study of particular microbes and pathways in broad environmental contexts. They reveal the cellular behaviors upon which an ecology is built. Yet the number of microbes, unique consortia and unique environmental contexts that exist represent a combinatorial explosion that is insurmountable to study purely *in vitro*. Further, *in vitro* technologies cannot capture the breadth of complexity within the host, and thus cannot integrate all relevant pathways, or reveal critical ecological parameters such as cellular nutritional state and competition dynamics, without disturbing or destroying the system. We have argued here that *in silico* approaches can span this divide. Genome-scale models can broadly supplement *in vitro* culturing efforts, though tuning and corroboration through *in vitro* means are necessary. *In silico* models enable observation and experimentation that reveal how cell-level behaviors underpin ecological processes to generate community-level outputs of relevance to the host (Figure 2). *In silico* techniques are undergoing rapid advancement and their full potential is as yet untapped. Efforts to functionally annotate genomes are accelerating, giving rise to increasing numbers of bacteria GSMs.^120^ Yet, their adoption is sparse relative to *in vitro* and *in vivo* experimental research.

There are aspects of the gut microbiome that influence health outcomes and are difficult to interrogate experimentally, but which could be amendable to study through computational modeling technologies. The non-bacterial (archaea, fungal, viral) gut microbiome is gaining recognition for its impact on host health,^7,8^ though such investigations are as yet scarce. Since *in silico* investigations rely on *in vitro-* and *in vivo-*derived data, exploration of the non-bacterial gut microbiome through computational modeling approaches will take some time to become established at scale. However, there is progress: GSMs of gut methanogenic archaea species have been added to AGORA^120^ and GSMs of (non-gut) protozoa can be found as part of the BiGG Models knowledgebase.^139^ Similarly, GSMs of fungal gut commensals and opportunistic pathogens have emerged in recent years.^140,141^ GSM has limited capacity to represent phages, however, as these viruses are not metabolically self-sufficient organisms. ABMs could represent the effects of phage-infection, and other behavior-modifying phenomena such as horizontal gene transfer (HGT). For instance, an ABM that explores bacteria-phage interactions at the community level and their effect on antibiotic resistance has already been developed,^142^ but this did not concern the gut microbiome specifically. Similarly, modeling efforts have been made toward the study of bacteria–bacteria HGT at the community level.^143–144^ Furthermore, ABM has scope for the study of spatiotemporal dynamics of mucosa-associated microbiome, offering some advantages over *in vitro* continuous fermentation models. For example, M-SHINE relied on static mucin-covered microcosms to replicate a mucin layer,^106^ whereas GutSim readily simulated real-world continuous mucin secretion patterns.^40^ We are unaware of any agent-based models that explicitly distinguish mucosal from luminal microbial communities, though it is not conceptually difficult to do so by, e.g., simulating an additional spatial dimension in GutSim. Detailed spatiotemporal analysis, beyond what is possible *in vivo*, would thus be possible.

Scientific investigations that integrate *in vitro, in vivo* and *in silico* perspectives are key to enable the knowledge that would move the field of personalized interventions forward. However, such efforts are exceedingly rare as research groups seldom possess deep capacity in all three. This is necessarily a broadly inter-disciplinary venture encompassing clinical and animal studies, anaerobic microbial culturing and bioengineering, omics, mathematics, and computer science. Dialog between these disciplines is not always easily established but should be encouraged, as should collaboration between research groups that specialize in these techniques. Education is already shifting to i) give biologists and health-care practitioners exposure to data and modeling, and ii) give a greater focus on clinical and biological application in engineering programs. The forthcoming convergence between disciplines together with our proposed integration of *in vivo, in vitro*, and *in silico* technologies will reveal the mechanistic underpinnings required for the design of rational interventions targeting the diet-gut microbiome-host system.

## List of abbreviations

SCFAs: Short-chain fatty acids

MAMPs: Microbe-associated molecular patterns

LPS: Lipopolysaccharide

SHINE: Simulator of the human intestinal microbial ecosystem

M-SHINE: Mucin-simulator of the human intestinal microbial ecosystem

ABM: Agent-based modeling/model

GSM: Genome-scale modeling/model

ARTIMMUS: artificial murine multiple-sclerosis simulation

MASON: Multiagent simulator of neighborhoods

COBRA: Constraint-based reconstruction analysis

KEGG: Kyoto encyclopedia of genes and genomes

AGORA: Assembly of gut organisms through reconstruction and analysis

HGT: Horizontal gene transfer
